# Oxidative Stress and Histomorphometric Remodeling: Two Key Intestinal Features of Type 2 Diabetes in Goto–Kakizaki Rats

**DOI:** 10.3390/ijms252212115

**Published:** 2024-11-12

**Authors:** Marisa Esteves-Monteiro, Mariana Ferreira-Duarte, Cláudia Vitorino-Oliveira, José Costa-Pires, Sara Oliveira, Paulo Matafome, Manuela Morato, Patrícia Dias-Pereira, Vera Marisa Costa, Margarida Duarte-Araújo

**Affiliations:** 1 Associated Laboratory for Green Chemistry (LAQV) Network of Chemistry and Technology (REQUIMTE), University of Porto, 4050-313 Porto, Portugalmmorato@ff.up.pt (M.M.); 2 Department of Immuno-Physiology and Pharmacology, Institute of Biomedical Sciences Abel Salazar, University of Porto (ICBAS-UP), 4050-313 Porto, Portugal; 3 Laboratory of Pharmacology, Department of Drug Sciences, Faculty of Pharmacy, University of Porto (FFUP), 4050-313 Porto, Portugal; 4 Institute for Health and Bioeconomy (i4HB), Laboratory of Toxicology, Department of Biological Sciences, FFUP, 4050-313 Porto, Portugalveramcosta@ff.up.pt (V.M.C.); 5 Research Unit on Applied Molecular Biosciences (UCIBIO), FFUP, Laboratory of Toxicology, Department of Biological Sciences, FFUP, 4050-313 Porto, Portugal; 6 Coimbra Institute for Clinical and Biomedical Research (iCBR) and Institute of Physiology, Faculty of Medicine, University of Coimbra (UC), 3000-548 Coimbra, Portugal; saraoliveira116@gmail.com (S.O.);; 7 Center for Innovative Biomedicine and Biotechnology (CIBB), UC, 3000-548 Coimbra, Portugal; 8 Clinical Academic Center of Coimbra (CACC), 3000-548 Coimbra, Portugal; 9 Coimbra Health School (ESTeSC), Polytechnic University of Coimbra, 3046-854 Coimbra, Portugal; 10 Department of Pathology and Molecular Immunology, ICBAS-UP, 4050-313 Porto, Portugal; pdiaspereira@yahoo.com.br

**Keywords:** diabetes, GK rats, gut remodeling, oxidative stress

## Abstract

Gastrointestinal complications of diabetes are often overlooked, despite affecting up to 75% of patients. This study innovatively explores local glutathione levels and morphometric changes in the gut of Goto–Kakizaki (GK) rats, a type 2 diabetes animal model. Segments of the intestine, cecum, and colon were collected for histopathological analysis and glutathione quantification. A significant increase in the total thickness of the intestinal wall of GK rats was observed, particularly in the duodenum (1089.02 ± 39.19 vs. 864.19 ± 37.17 µm), ileum (726.29 ± 24.75 vs. 498.76 ± 16.86 µm), cecum (642.24 ± 34.15 vs. 500.97 ± 28.81 µm), and distal colon (1211.81 ± 51.32 vs. 831.71 ± 53.2 µm). Additionally, diabetic rats exhibited thickening of the muscular layers in all segments, except for the duodenum, which was also the only portion where the number of smooth muscle cells did not decrease. Moreover, myenteric neuronal density was lower in GK rats, suggesting neurological loss. Total glutathione levels were lower in all intestinal segments of diabetic rats (except duodenum), and the reduced/oxidized glutathione ratio (GSH/GSSG) was significantly decreased in GK rats, indicating increased oxidative stress. These findings strongly indicate that GK rats undergo significant intestinal remodeling, notable shifts in neuronal populations, and heightened oxidative stress—factors that likely contribute to the functional gastrointestinal alterations seen in diabetic patients.

## 1. Introduction

Diabetes is a highly prevalent metabolic disorder characterized by a state of hyperglycemia [1]. The most recent data from the International Diabetes Federation indicate that diabetes affected 537 million people worldwide in 2021, a number that is expected to grow to 643 million by 2030 [2]. Besides the substantial economic impact of the disease, the importance of diabetes is also related to significant mortality and morbidity rates, being considered a major public health problem [3,4,5]. There are two main forms of diabetes: type 1 diabetes (T1D) and type 2 diabetes (T2D). T1D is caused by an absolute insulin deficiency while T2D is a combination of insulin resistance in target organs and relative deficiency caused by dysfunctional pancreatic β-cells [6]. T2D is far more prevalent, accounting for 90 to 95% of all cases [7,8]. Around 80% of adult T2D patients are considered overweight or obese. However, 10–15% are not obese, and those patients present higher hypoglycemic events and mortality rates [9]. Given the significant importance and wide prevalence of diabetes, numerous animal models are employed in the study of diabetes-related complications [10]. The Goto–Kakizaki (GK) rat is a non-obese animal model of T2D that was developed by Goto, Kakizaki, and Masaki in 1975 [11,12]. This model was obtained by selective reproduction of non-diabetic Wistar rats with slight glucose intolerance. Consequently, the rats from posterior generations spontaneously developed T2D without becoming obese [13,14]. GK rats exhibit a reduced pancreatic β-cell number and function, moderate hyperglycemia, glucose intolerance, and peripheral insulin resistance [15].

Diabetes frequently concurs with gastrointestinal (GI) complications that are associated with significant morbidity, affecting up to 75% of patients. Currently, it is unclear whether the prevalence differs between T1D and T2D [16]. In the small intestine and colon, diabetes-related complications usually result in symptoms like chronic constipation, diarrhea, and fecal incontinence that may result in potential complications such as megacolon, pseudo-obstruction, stercoral ulcer, and perforation [17,18]. In addition, diabetes seems to worsen clinical conditions such as colorectal cancer and inflammatory bowel disease [19]. Although highly prevalent, these symptoms are often overlooked, as they do not significantly contribute to mortality in diabetic patients. However, it is crucial to acknowledge that they negatively impact health status and quality of life, making them a significant source of morbidity [20]. The relationship between diabetes and the pathogenesis of the described gut disorders is not completely understood and seems to be multifactorial [21]. Mechanical factors contribute to intestinal disorders, since it has been found that diabetes seems to cause structural remodeling that can affect histomorphometry and biomechanical properties, increase stiffness, and decrease the resting compliance and relaxation capacity of the intestinal wall [21]. A previous study by our group also showed significant histomorphometry changes and evidenced lower reactivity to angiotensin II of the ileum and colon of T1D-induced rats [22]. Also, Zhao et al. demonstrated the existence of remodeling in the esophagus and stomach of GK rats [23], while Pereira et al. showed alterations of the small intestine in the same animal model [24]. So far, only one study has shown colon remodeling in a T2D model, associating it with the formation of advanced glycation end products [25].

In diabetes, various pathways contribute to tissue damage, but a common hallmark is heightened oxidative stress, characterized by elevated levels of reactive oxygen species (ROS) [26]. Moreover, chronic hyperglycemia is linked to decreased cellular levels of glutathione (GSH) [27]. GSH is the most powerful intercellular antioxidant in an organism, undergoing oxidation to GSSG (glutathione disulphide or oxidized glutathione) after contact with electrophiles. These reactions can be catalyzed by GSH-peroxidase. GSSG can subsequently be regenerated back to GSH by GSH-reductase, using NADPH as a cofactor, or it is excluded from the cell through membrane transporters (e.g., multidrug resistance-associated proteins, MRPs). Maintaining an optimal ratio of GSH to GSSG within the cell is crucial for survival, and a decrease in this ratio may be used as a marker of oxidative stress [28]. Oxidative stress and ROS formation have already been described to be markedly increased by uncontrolled hyperglycemia [29]. Also, a decrease in GSH has been observed in the liver [30], erythrocytes [31], and colon [32] of long-term diabetic patients. But so far, there are no data regarding GSH local levels in diabetic small intestines.

Curiously, most researchers studying diabetes-related complications in the GI tract use animal models of T1D, even though T2D is the most common form [21]. Considering this, and that diabetic patients commonly present GI complications, we innovatively aimed to characterize the entire gut histomorphometry and the local glutathione system in an animal model of T2D. Examining the entire gut—from the duodenum to the distal colon—in the same animals allows for a direct comparison between segments, providing a clearer understanding of how diabetes uniquely affects each part of the gastrointestinal tract. Additionally, local glutathione levels offer a more precise picture than systemic levels because they provide insights into the specific redox environment and oxidative stress within a targeted tissue or organ—like the gut in this case. Systemic GSH levels represent an overall average throughout the body, which can mask localized changes or stresses. In contrast, studying local GSH concentrations allows us to understand how the oxidative balance is maintained or disrupted in a specific region, which is particularly relevant for organs impacted by T2D, where localized oxidative stress can contribute to disease progression. To achieve this goal, we took samples of GK rats’ duodenum, middle jejunum, distal ileum, cecum, and proximal and distal colon and measured the individual layers of the intestinal wall, analyzed smooth muscle cells and myenteric neurons, and quantified GSH and GSSG levels.

## 2. Results

### 2.1. Animal Monitorization and Insulin Tolerance Test

**Figure 1:**
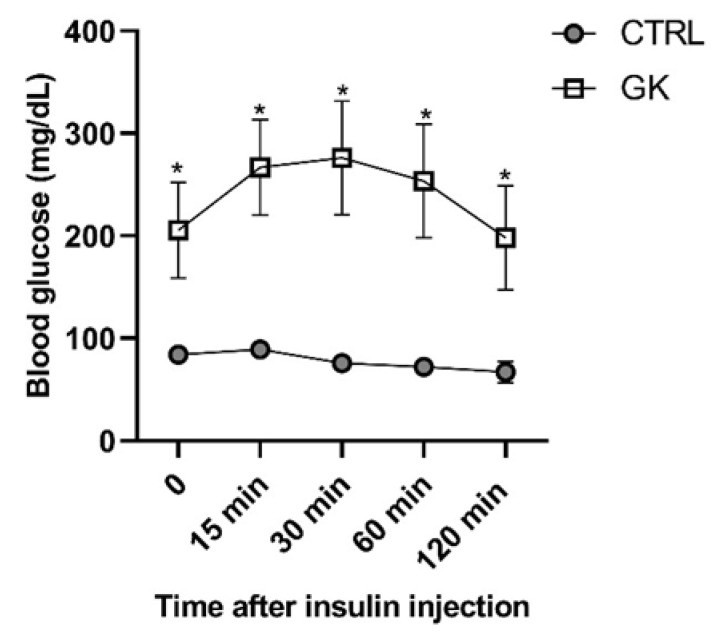
Blood glucose concentrations of control (CTRL, *n* = 5) and GK animals (*n* = 6) measured before (time 0) and during the insulin tolerance test—ITT. Values are presented as mean ± SEM, and a paired Student’s *t* test was used to compare the two experimental groups (CTRL and GK). * Statistical difference, *p* < 0.05.

GK rats presented elevated fasted glucose concentrations compared to controls (237.88 ± 81.05 mg/dL vs. 100 ± 1.73 mg/dL, respectively, *p* < 0.05) (time 0, Figure 1). After a 6 h fasting and insulin administration, the glycemia of the GK group increased during the first 30 min and then decreased, reaching the initial glycemic quantification at the end of the insulin tolerance test (ITT, time 120 min, Figure 1). In the control group, after insulin injection a slight decline in blood glucose values was observed. Compared to the control (CTRL) group, the GK group’s blood glucose concentration was higher at all time points (*p* < 0.0001, Figure 1).

**Figure 2:**
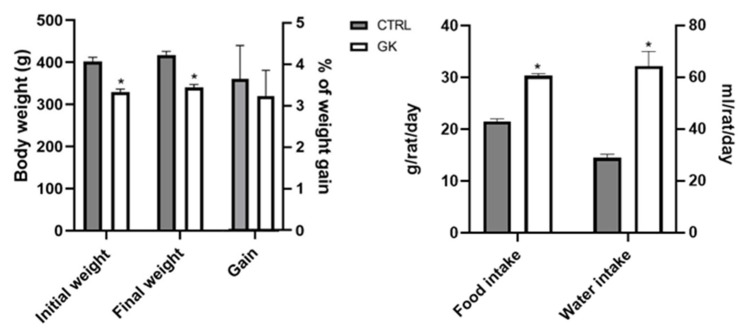
Evaluation during the experimental protocol of control (CTRL, *n* = 5) and GK diabetic rats (GK, *n* = 6) of: body weight; body weight gain; food intake and water intake. Values are presented as mean ± SEM and unpaired Student’s *t* test was used to compare the two experimental groups (CTRL and GK). * Statistical difference, *p* < 0.05.

At the beginning of the protocol, the weight of the GK group was on average 329.17 ± 7.4 g, increasing 2 weeks later to 340.17 ± 6.95 g, representing an average weight gain of 3.24 ± 0.62%. The control rats weighed 402.20 ± 9.56 g at the beginning of the protocol and 417.40 ± 8.81 at the end, representing an average weight gain of 3.65 ± 0.81% (Figure 2). So, the initial and final weights of GK rats were both lower compared to controls (*p* < 0.0001), but the % of weight gain during the experimental period was roughly the same in the two groups (*p* > 0.05) (Figure 2). Despite maintaining the same amount of weight gain as controls, the food intake of GK rats (28.95 ± 1.40 mg/day/rat) was significantly higher than that of controls (21.50 ± 0.50 mg/day/rat) (Figure 2). Regarding water intake, it was significantly higher in diabetic rats compared to controls (Figure 2). The GK group drank 64.38 ± 5.63 mL/day/rat (*n* = 6), which was more than double the water that control animals drank (30.30 ± 0.40 mL/day/rat, *n* = 5).

### 2.2. Small Intestine and Colon Microscopic Evaluation

**Figure 3:**
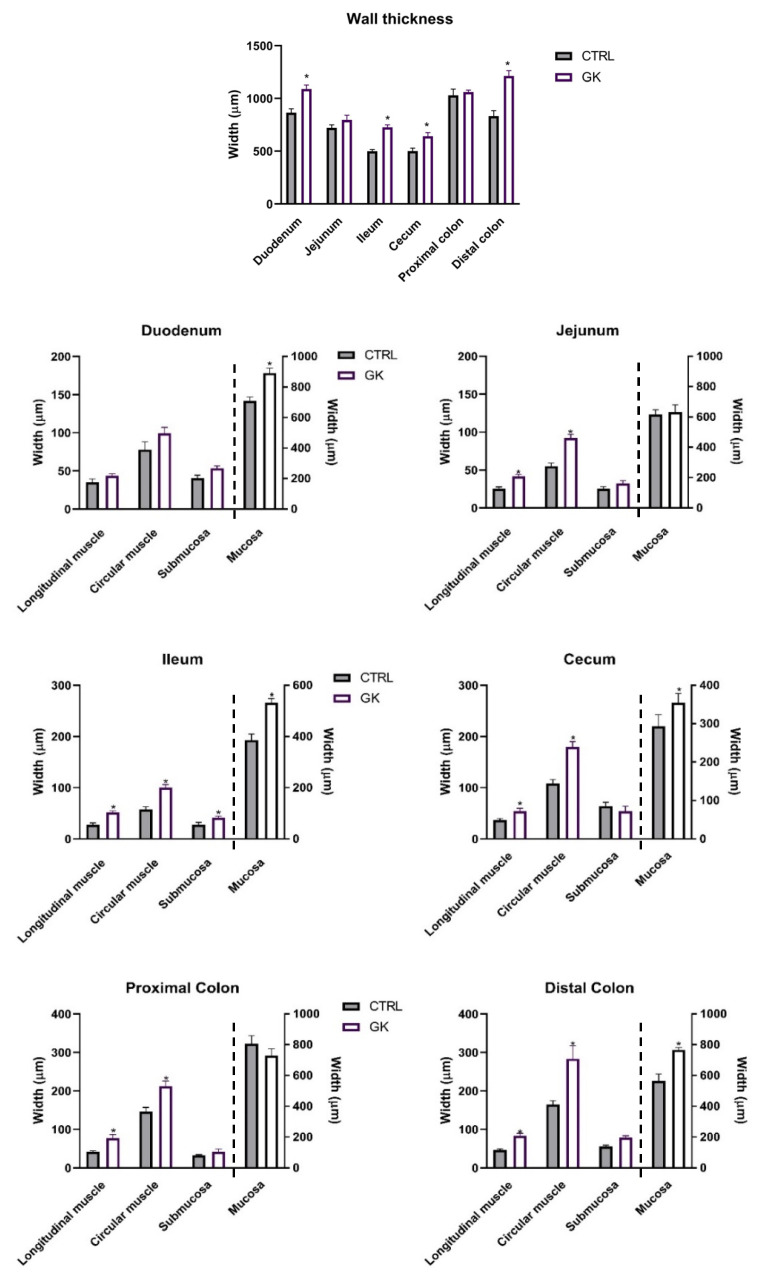
Morphometric evaluation of intestinal segments (duodenum, jejunum, ileum, cecum, proximal colon, and distal colon) of control (CTRL, *n* = 5) and GK diabetic rats (GK, *n* = 6): total wall thickness (μm) of each intestinal segment and thickness (μm) of the intestinal layers (longitudinal muscle, circular muscle, submucosa, and mucosa) of duodenum, jejunum, ileum, cecum, proximal colon, and distal colon). Values are presented as mean ± SEM, and a 2-way ANOVA followed by an unpaired *t* test with Welch’s correction was used to compare the two experimental groups (CTRL and GK). * Statistical difference *p* < 0.05 vs. correspondent control. Unpaired *t* test with Welch’s correction was used to compare the two experimental groups.

To assess whether gut remodeling occurs and follows a proximal-to-distal progression, as previously observed in T1D rat models, we measured both the mucosal and muscle layers in a T2D rat model. The histomorphometric evaluation of the small and large intestine of GK animals showed a higher thickness of the total intestinal wall of the duodenum, ileum, cecum, and distal colon (DC) compared to controls (duodenum: 1089.02 ± 39.19 µm vs. 864.19 ± 37.17 µm; ileum: 726.29 ± 24.75 µm vs. 498.76± 16.86 µm; cecum: 642.24 ± 34.15 µm vs. 500.97 ± 28.81 µm; DC: 1211.81 ± 51.32 µm vs. 831.71 ± 53.25 µm, respectively, *p* < 0.01 for all). There was no difference between GK and control rats in a histomorphometric evaluation of the jejunum and proximal colon (PC) (jejunum: 796.16 ± 43.86 µm vs. 722.12 ± 28.75 µm and PC: 1060.18 ± 18.93 µm vs. 1029.01 ± 59.84 µm, respectively, *p* > 0.05 for both) (Figure 3).

The muscular layers of the intestinal wall of GK animals were increased in all segments except in the duodenum compared to controls (jejunum—longitudinal muscle (lm): 41.69 ± 2.80 µm vs. 25.54 ± 2.28 µm, circular muscle (cm): 91.99 ± 5.03 µm vs. 55.33 ± 3.73 µm; ileum—lm: 51.99 ± 2.90 µm vs. 27.87 ± 3.14 µm, cm: 100.11 ± 5.96 µm vs. 57.19 ± 5.38 µm; cecum—lm: 54.44± 5.33 µm vs. 36.57 ± 3.15 µm, cm: 179.36 ± 10.84 µm vs. 107.82 ± 8.09 µm; PC—lm: 77.70 ± 8.97 µm vs. 42.52 ± 1.87 µm, cm: 212.03 ± 13.73 µm vs. 146.03 ± 11.12 µm; DC—lm: 83.31 ± 6.54 µm vs. 46.04 ± 3.51 µm, cm: 283.40 ± 33. 86 µm vs. 164.43 ± 3.51 µm, respectively, *p* < 0.05 for all; duodenum—lm: 43.81 ± 2.67 µm vs. 35.12 ± 4.30 µm, cm: 99.36 ± 7.80 µm vs. 78.08 ± 9.93 µm, respectively, *p* > 0.05) (Figure 3). Submucosal values were consistent across all portions, except for the ileum in GK rats, where an increase was observed (GK: 41.73 ± 2.9 µm vs. CTRL: 28.04 ± 4.38 µm). The mucosa was only increased in the duodenum (GK: 892.48 ± 31.21 µm vs. CTRL: 710.60 ± 24.82 µm), ileum (GK: 532.46 ± 15.87 µm vs. CTRL: 385.66 ± 24.20 µm), and DC (GK:765.84 ± 16.86 µm vs. CTRL: 566.01 ± 44.33 µm) of GK rats compared with controls, while the jejunum (GK: 630.34 ± 49.26 µm vs. CTRL: 615.97 ± 30.80 µm), cecum (GK: 354.70 ± 24.00 µm vs. CTRL: 292.72 ± 30.77 µm), and PC (GK: 728.53 ± 45.79 µm vs. CTRL: 808.19 ± 51.10 µm) presented similar results in both GK and control animals (Figure 3). Additionally, in the epithelial layer, villi length and crypt depth were also increased in the duodenum and ileum of GK rats (Supplementary Materials, Figure S1).

**Figure 4:**
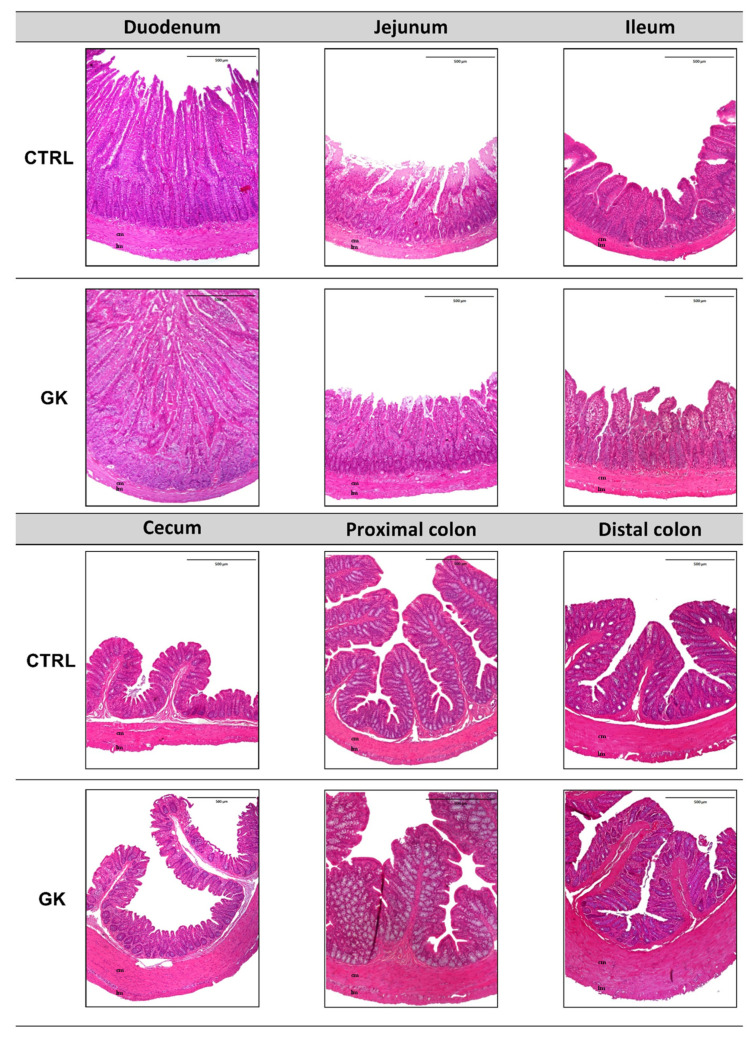
Representative microscopic photographs of duodenum, jejunum, ileum, cecum, proximal colon, and distal colon of control (CTRL) and GK rats (GK) stained with hematoxylin (blue) and eosin (pink), captured using 40× magnification. Longitudinal muscle (lm) and circular muscle (cm) were identified in all images.

In Figure 4, representative images of both control (CTRL) and GK animals are displayed, encompassing all the studied sections. These images provide a comprehensive visual comparison, highlighting the differences in each portion analyzed.

**Figure 5:**
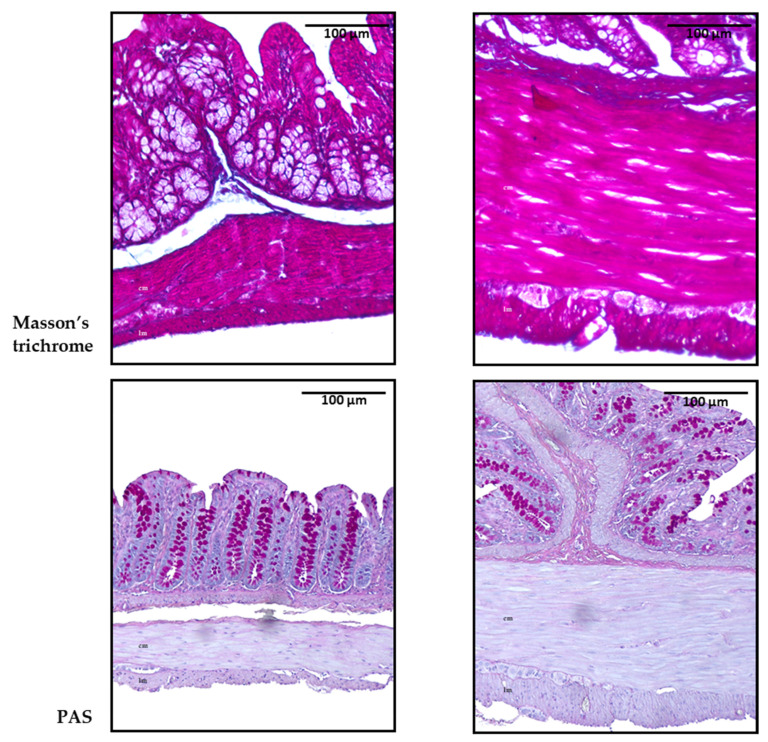
Representative microscopic photographs of the colon of control (CTRL) and GK rats (GK) stained with Masson’s trichrome and periodic acid–Schiff (PAS), captured at 100× magnification. Longitudinal muscle (lm) and circular muscle (cm) were identified in all images.

Then, collagen deposition was measured to evaluate potential tissue remodeling and fibrosis, conditions commonly linked to chronic hyperglycemia. These factors could explain the increased thickness of the muscular layers observed in the histomorphometric analysis. Masson’s trichrome and periodic acid–Schiff (PAS) stains were assessed by an experienced pathologist blinded to the experiments. Interestingly, qualitative evaluation revealed no discernible differences between the control and GK diabetic animals. This suggests the absence of collagen deposition and no meaningful disparity in the proportion of carbohydrate macromolecules, such as glycogen, between the GK and control animal groups. Representative microscopic photographs of the colon with both staining techniques are shown in Figure 5.

### 2.3. Smooth Muscle Cell Density in the Muscular Layers

**Figure 6:**
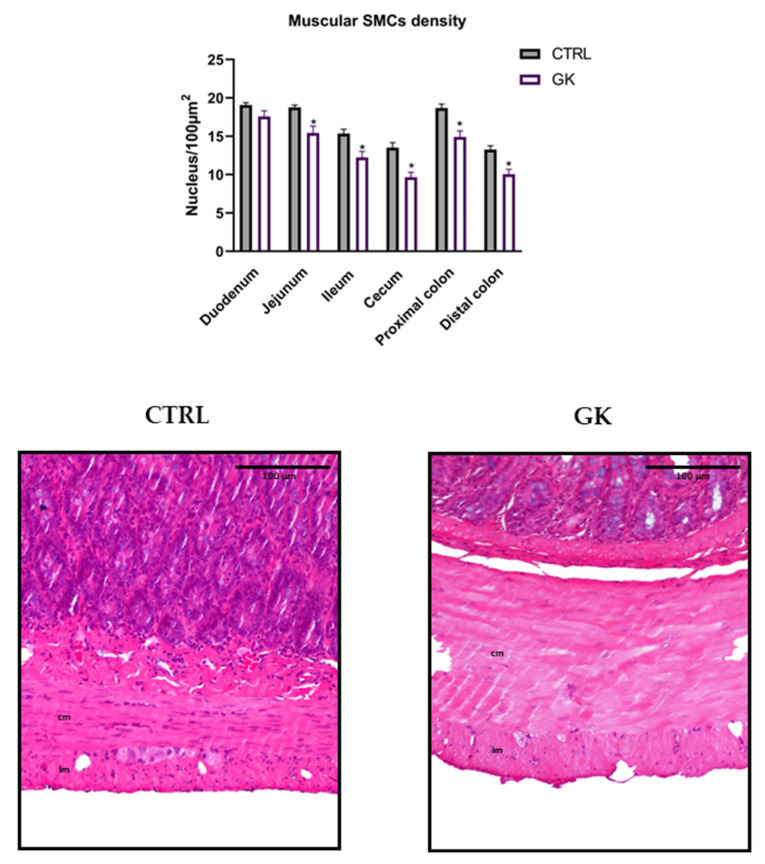
Morphoquantitative analyses of the density of smooth muscle cells (SMCs) in the muscular layers of duodenum, jejunum, ileum, cecum, and proximal and distal colon of control group (CTRL, *n* = 5) and GK diabetic rats (GK, *n* = 6). Data are expressed as the mean ± SEM, and comparisons between the two groups were made using Student’s *t* test. * Statistical difference, *p* < 0.05. Representative microscopic photographs of the muscle layers of distal colon of control (CTRL) and GK rats (GK) stained with hematoxylin and eosin, captured at 100× magnification. Longitudinal muscle (lm) and circular muscle (cm) were identified in both images.

Smooth muscle cell density was quantified in response to the negative results from Masson’s trichrome staining to determine whether an increase in density might be linked to smooth muscle hypertrophy. The number of nuclei of smooth muscle cells (SMCs) was lower in GK rats in all portions studied except for the duodenum compared to controls (jejunum: 15.42 ± 0.89 vs. 18.75 ± 0.1; ileum: 12.23 ± 0.80 vs. 15.35 ± 0.57; cecum: 9.65 ± 0.65 vs. 13.50 ± 0.67; PC: 14.90 ± 0.80 vs. 18.68 ± 0.52; DC: 10.06 ± 0.64 vs. 13.25 ± 0.51, respectively, *p* < 0.02 for all; duodenum: 17.58 ± 0.74 vs. 19.08 ± 0.31, respectively, *p* > 0.05). Representative microscopic images focusing on the muscular layers are depicted in Figure 6.

### 2.4. Neuronal Density in the Myenteric Plexi

Neuron density was also assessed, based on the findings of Honoré et al. (2011), which suggested that neuronal loss could contribute to increased colonic thickness, potentially due to the greater force required for motility. Both smooth muscle cell and neuron densities are critical for maintaining proper gastrointestinal motility and function, which may be compromised by prolonged diabetes.

**Figure 7:**
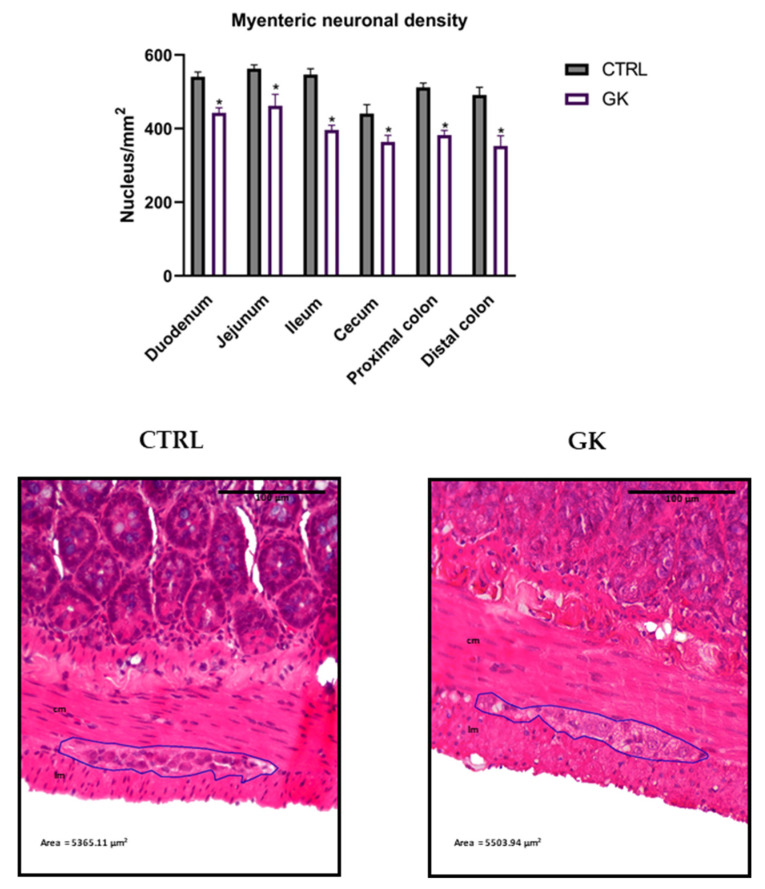
Morphoquantitative analyses of the neuronal density in the myenteric plexus of duodenum, jejunum, ileum, cecum, and proximal and distal colon of control group (CTRL, *n* = 5) vs. GK diabetic rats (GK, *n* = 6). Data are expressed as the mean ± SEM, and comparisons between the two groups were made using Student’s *t* test. * Statistical difference, *p* < 0.05. Representative microscopic photographs of the myenteric plexus proximal colon of control (CTRL) and GK rats (GK) stained with hematoxylin and eosin, captured at 100× magnification. Longitudinal muscle (lm) and circular muscle (cm) were identified in both images.

The neuronal density in the myenteric plexus was lower in the GK group, when compared to control rats (Figure 7). The number of nuclei per mm^2^ was statistically lower in diabetic animals compared to controls in all portions studied (duodenum: 444.95 ± 13.97 vs. 540.54 ± 21.47; jejunum: 461.65 ± 31.78 vs. 562.62 ± 10.86; ileum: 396.36 ± 12.73 vs. 546.63 ± 15.94; cecum: 363.81 ± 17.74 vs. 440.65 ± 24.82; PC: 382.36 ± 12.34 vs. 511.90 ± 11.85; DC: 352.65 ± 27.94 vs. 491.03 ± 21.47, respectively, *p* < 0.05 for all). Representative microscopic images focusing on the myenteric plexus are depicted in Figure 7.

### 2.5. Total GSH and GSSG Quantification

**Figure 8:**
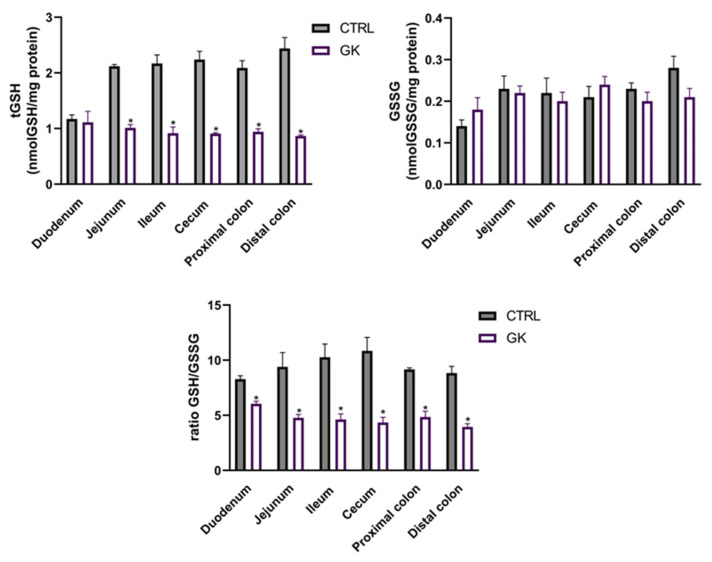
Glutathione evaluation of intestinal segments (duodenum, jejunum, ileum, cecum, proximal colon, and distal colon) of control (CTRL, *n* = 5) and GK diabetic rats (GK, *n* = 6): total glutathione (tGSH) quantification (nmol GSH/mg protein); oxidized glutathione (GSSG) quantification (nmol GSSG/mg protein) and ratio GSH/GSSG. Values are mean ± SEM, and an unpaired Student’s t test with Welch’s correction was used to compare the two experimental groups (CTRL and GK). * Statistical difference *p* < 0.05 vs. correspondent control.

To investigate the potential causes of decreased neuron density observed in the myenteric plexus of GK rats, we decided to measure GSH levels as an indicator of oxidative stress, a critical factor in the development of diabetic complications. The results of the total glutathione quantification showed a decrease in tGSH in diabetic animals compared to controls in all portions studied except the duodenum (in nmol tGSH/mg protein, jejunum: 1.01 ± 0.06 vs. 2.11 ± 0,03; ileum: 0.92 ± 0.11 vs. 2.17 ± 0,15; cecum: 0.91 ± 0.01 vs. 2.24 ± 0.15; PC: 0.94 ± 0.06 vs. 2.09 ± 0.12; DC: 0.87 ± 0.02 vs. 2.44 ± 0.19, respectively, *p* < 0.02 for all; duodenum: 1.11 ± 0.20 vs. 1.17 ± 0.07, respectively, *p* > 0.05). However, the quantification of GSSG revealed comparable values between GK rats and controls across all studied portions (*p* > 0.05 for all) (Figure 8). Regarding the GSH/GSSG ratio, a decrease was observed in all portions of GK diabetic rats compared to controls (duodenum: 6.04 ± 0.24 vs. 8.28 ± 0.32; jejunum: 4.77 ± 0.31 vs. 9.39 ± 1.31; ileum: 4.62 ± 0.52 vs. 10.27 ± 1.20; cecum: 3.94 ± 0.31 vs. 10.84 ± 1.22; PC: 4.84 ± 0.53 vs. 9.15 ± 0.16; DC: 4.35 ± 0.47 vs. 8.83 ± 0.62, respectively, *p* < 0.05 for all) (Figure 8).

## 3. Discussion

This study presents a novel approach to examining local glutathione levels and morphometric changes in the entire gut of GK rats. To the best of our knowledge, this is the first comprehensive study to analyze histomorphometry and quantify tGSH, GSSG, and the GSH/GSSG ratio across the entire gut, including the proximal and distal colon, all intestinal segments, and the cecum in a T2D model. The assessment of local GSH provides a more localized and precise evaluation of oxidative stress within the gastrointestinal tract. This is a novel approach in diabetic animal models, offering new insights into how different regions of the intestine respond to diabetes-related oxidative stress.

In this work, 21-week-old male GK rats exhibited reduced weight compared to their Wistar counterparts while presenting higher food intake. They also showed fasting basal hyperglycemia and impaired insulin sensitivity when compared to the control group. It has already been shown that GK animals fail to accumulate body fat despite their higher calorie consumption and that adipose tissue is a major contributor to the differential weight in these animals [33]. These changes are due to an impairment in pre-adipocyte differentiation into mature adipocytes, leading to a defect in triglyceride storage [33]. Therefore, these findings align with the expectations for the GK model, since the average body weight of a GK rat is expected to be 10–30% less than that of an age-matched control Wistar rat [34]. Basal hyperglycemia has also been documented in GK rats, often manifesting as early as 3 weeks of age [35]. At birth, the β-cell mass of a GK rat is already severely reduced compared to that of a Wistar rat [36], and in adult GK rats, the β-cell mass is usually reduced up to 60% with markedly decreased insulin secretion [37,38], which explains the early hyperglycemia. Insulin resistance, another well-documented trait of this genetic model of T2D [15,39], also aligns with the findings of our study. Although inadequate β-cell proliferation in early life is a limitation as it relates to the human condition, other characteristics are consistent with descriptions in the literature and validate GK rats as a non-obese T2D animal model [34,40].

Histomorphometric changes in the gut of other animal models of diabetes have already been described [21,22,24,41,42], but this is the first study to comprehensively examine the gut from the duodenum to the distal colon. This approach was chosen in order to ascertain whether we would observe a similar proximal-to-distal progression of the disease as previously described in models of T1D [22,43]. In the GK rat, a T2D model, we did not observe such a pattern. Gut remodeling appears to occur in both mucosa and muscle layers, in different regions of the gut. The increase in the mucosa layer has been reported, and it was suggested that it is a mechanism to augment the absorptive surface area and functional capacity of the intestine [44]. Hyperphagia occurs in almost every model of diabetes and has also been suggested as a contributor to the increase in the thickness of intestinal mucosa [43]. However, it appears that the hyperglycemic state itself is sufficient to promote significant mucosal growth independent of food intake [45]. This was reinforced by another study where insulin administration prevented a marked increase in the intestinal epithelial cell proliferation rate of type 1 diabetic rats, resulting in reduced intestinal mucosal growth compared to non-treated diabetic animals [46]. Adachi et al. showed that GK rats also exhibited intestinal hyperplasia, possibly due to the increased expression of transcription factors and proteins involved in cell regeneration, differentiation, and/or proliferation [47]. An increase in the muscle layers of the gut has also already been reported in several animal models of diabetes [21,24,41,48]. In our study, the increase in the thickness of muscular layers may be at least partially attributed to hypertrophy of SMCs since we observed a decreased density, rather than an increase. This finding was consistent across all portions examined (not reaching statistical significance in the duodenum). SMC hypertrophy has already been described by Horváth et al., who associated this alteration with contractile protein actin and myosin increases in diabetic patients [17].

The myenteric plexus is located between the circular and the longitudinal muscular layers and is the main thing responsible for GI motility control [16,49]. In contrast to the findings of Pereira et al. [24], who did not observe a significant difference in the number of myenteric neurons per unit area between GK animals and controls, our study revealed a decrease in the density of myenteric neurons in diabetic animals. It is worth noting that our animals were older compared to those in the study by Pereira et al. [24]. Therefore, the duration of diabetes may play a role in the development of these alterations. Additionally, several authors also reported changes in the number and size of myenteric neurons throughout the entire GI tract, including the stomach [43], duodenum [50], jejunum [51], ileum [49], cecum [52], and colon [53], in both type 1 (streptozotocin-induced diabetic rats) [54] and type 2 D (diabetic mice consuming a high-fat diet) [55]. It seems that the neuronal population of the submucosal plexus may be more susceptible to degenerative changes induced by diabetes than the myenteric plexus [56]. The mechanisms underlying neuronal loss encompass increased apoptosis, elevated levels of Advanced Glycation End products (AGEs) and Receptor of Advanced Glycation End products (RAGEs), reduced nerve growth factor levels, and heightened oxidative stress [51,53,57].

These changes in the morphology of the small intestine and colon result in biomechanical alterations such as a loss of matrix elasticity and contractility, impairing both contraction and relaxation responses, which are fundamental for maintaining normal GI motility [32,58]. This leads to impaired intestinal sensory function and reduced intestinal motility [41,59,60], while increased thickness of the mucosa can affect digestion and absorption [61]. The neuronal change can further lead to improper gut motility, retrograde colonic movements, altered secretions, and even increased pain stimuli [62,63]. These alterations may provide insight into the common GI symptoms observed in diabetic patients [21].

Oxidative stress results from an imbalance between the production of ROS and antioxidant defenses and has already been implicated in gastrointestinal complications of diabetes [64,65]. Given that glutathione serves as the body’s primary antioxidant, playing a crucial role in combating oxidative stress [66], and that previous studies showed that hyperglycemia-related oxidative stress was a primary inducer of neurological damage [16], we chose to quantify GSH levels locally. To the best of our knowledge, this is the first study to comprehensively evaluate tGSH and GSSG levels and the GSH/GSSG ratio across all sections of the gut in diabetic animals. In this work, we observed a decrease in tGSH levels in all examined segments of the gut, except for the duodenum. Furthermore, while the levels of GSSG were comparable between diabetic and control animals, the ratio of GSH to GSSG was significantly lower in diabetic animals (including in the duodenum), indicating increased levels of oxidative stress. The reduction in the GSH/GSSG ratio can result from either a decrease in free GSH levels or an increase in GSSG levels. In this study, the observed decrease in the GSH/GSSG ratio in GK rats was primarily due to a reduction in GSH levels in GK animals compared to controls, as there were no significant differences in GSSG levels between the two experimental groups. Chandrasekharan et al. conducted the first and only quantification of GSH but only in the diabetic colon as an indicator of oxidative stress, wherein they also observed a decrease in GSH levels associated with neurological damage and motor dysfunction [32]. These are likewise consistent with findings in individuals with T2D, who have been reported to exhibit lower blood GSH values [67,68]. Also, the depletion of GSH observed in a streptozotocin-induced model of diabetes has been shown to cause cardiac damage and cardiomyocyte apoptosis [69]. In another study, a decrease in GSH levels was observed in vascular smooth muscle cells, which was attributed to the depletion of glutathione precursors, particularly cysteine, which is a rate-limiting substrate in new glutathione synthesis [70]. Sekhar et al. described that the principal cause of oxidative stress in T2D is a deficiency of glutathione, primarily stemming from reduced synthesis due to the limited availability of the precursor amino acids cysteine and glycine, and that the supplementation of these precursors through dietary means can restore the synthesis of glutathione, consequently leading to a significant reduction in oxidative stress and markers of oxidant damage [71]. Furthermore, in individuals with type 2 diabetes, increased levels of transforming growth factor beta (TGF-β) were observed in plasma samples. This cytokine is known to reduce the expression of the catalytic subunit of glutamine-cysteine ligase, which also helps to explain why GSH levels decrease in these individuals [72].

The alterations observed in the GSH and GSSG concentrations in our study led to a decrease of up to 60% in the GSH/GSSG ratio in GK rats compared to controls. This reduction closely mirrors findings reported by Calabrese et al., who observed a 68% decrease in plasma GSH levels in T2D patients compared to control subjects [73]. The decrease in the plasma GSH/GSSG ratio not only correlates with heightened oxidative stress but also appears to adversely affect glucose availability and homeostasis, thereby exacerbating the diabetic condition [74,75]. Additionally, oxidative stress is known to play a critical role in the pathogenesis of various diabetic complications, including neuropathy, nephropathy, and retinopathy [27]. Furthermore, oxidative stress has also been identified as a significant contributor to gastrointestinal dysmotility, including post-operative ileus and diabetic gastroparesis [64]. Maintaining a balanced GSH/GSSG ratio is essential for protecting cells from oxidative damage and ensuring proper metabolic functioning [76]. Therefore, our findings highlight the importance of addressing oxidative stress when studying gastrointestinal complications of diabetes.

The results of histomorphometry and oxidative stress combined reveal an interesting pattern: all sections of the intestine showed signs of oxidative stress (indicated by a decreased GSH/GSSG ratio) and neuronal damage, but muscular remodeling was not observed in every portion. In fact, the duodenum displayed both oxidative stress and neuronal damage, but the muscular layers showed no remodeling. This raises an important question: does neuronal damage from oxidative stress occur before intestinal remodeling? These findings prompt further investigation into the sequence of events leading to gastrointestinal complications in diabetes.

**Figure 9:**
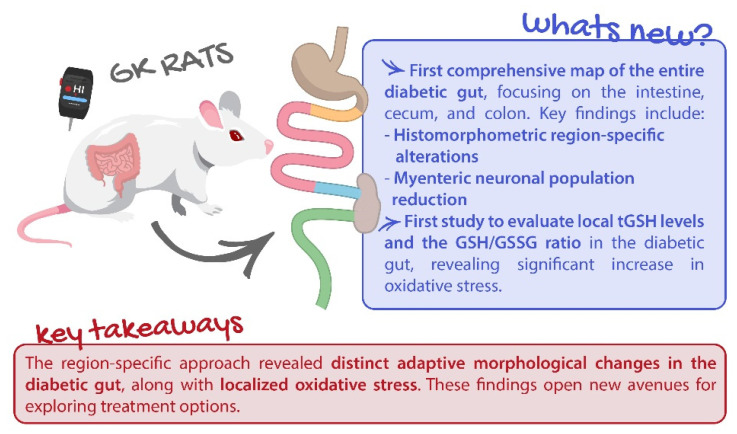
This study combines histomorphometry with glutathione assessments, providing a dual layer of analysis that allows for a more comprehensive understanding of tissue health and oxidative damage across different diabetic gut regions.

In conclusion, we identified significant remodeling of the intestine and colon, along with marked alterations in the neuronal population of the myenteric plexus. The critical local deficiency of GSH, a key antioxidant, emerged as a central factor driving increased oxidative stress, which likely underlies the observed structural and neuronal damage in the gut of GK rats. Furthermore, the reduced GSH/GSSG ratio further underscores the oxidative stress in the examined gut regions. These data shed some light on the complex interplay between diabetes and gastrointestinal adjustments, offering new insights that could enhance our understanding and management of diabetic complications (Figure 9).

## 4. Materials and Methods

### 4.1. Animals

Non-obese type 2 diabetic GK male rats (*n* = 6), 20–21 weeks old, were obtained from the breeding colonies of the Faculty of Medicine, University of Coimbra. Wistar Han rats (*n* = 5) from the same colony with comparable age were used as controls. Animals were kept under standard ventilation, temperature (22.0 ± 0.1 °C), relative humidity (52.0 ± 2.0%), and light (12 h light/dark cycle) with access to autoclaved tap water and food ad libitum (standard diet A03, SAFE^®^, Rosenberg, Germany). All procedures involving animals were previously approved by the local animal welfare commission (ORBEA 13/18) following the European Community guidelines for the use of laboratory animals (Directive 2010/63/EU) and performed by licensed users.

### 4.2. In Vivo Procedures and Sample Collection

Animals’ body weight, caloric intake, and blood glucose (6 h fast, blood collected from the tail vein) were monitored for 2 weeks.

Intraperitoneal insulin tolerance tests (ITTs, Humulin, Lilly^®^, Indianapolis, Indiana, USA, 0.25 IU/kg) were performed after a 6 h fast. Glycaemia evaluation was performed at 0, 15, 30, 60, and 120 min using a glucometer and test strips (Accu-Chek Aviva, Roche^®^, Basel, Switzerland) [77].

After a 6 h fast, animals were anesthetized with an intraperitoneal injection of ketamine (Nimatek, Dechra^®^, Northwich, England, 50 mg/kg) and xylazine (Sedaxylan, Dechra^®^, 6.6 mg/kg) and after blood collection were sacrificed by cervical displacement. The GI tract from the proximal part of the duodenum to the distal part of the colon was collected and weighed as previously described by our group [22].

### 4.3. Histological Preparation and Analyses

Samples (1 cm long) of proximal duodenum (collected 2 cm distal to pylorus), middle jejunum, distal ileum (collected 2 cm cranial to the ileocecal junction), cecum, proximal colon (PC), and distal colon (DC) were collected and fixed in 4% formalin. All samples were dehydrated in consecutive 70%, 96%, and 99% ethanol solutions and embedded in paraffin. Then, 3 µm thick cuts were made perpendicularly to the mucosa using a microtome and mounted in sterilized glass slides. Finally, the sections were rehydrated in a series of graded ethanol (99, 96, 70%), washed in water, and stained with hematoxylin and eosin (H&E). Each section was evaluated under an optical microscope (Eclipse E600Miami, Nikon Instruments^®^, Melville, NY, USA) and photographed in different representative regions (magnification of 40× and 100×). All stained samples were evaluated by an experienced veterinary pathologist who was blinded for the experimental groups. The thickness of the mucosa, submucosa, circular, and longitudinal muscles was then measured, by the same research team member, using the free ImageJ^®^ software 1.54g. For each sample, the layer thickness was measured randomly in twelve different locations, and then averaged. The measurements were only carried out for images where the entire intestinal wall could be observed. To evaluate collagen deposition in the extracellular matrix, the samples were stained with Masson’s trichrome, and to measure the intracellular accumulation of glycogen, the periodic acid–Schiff (PAS) reaction was performed. All histologic samples were evaluated by an experienced veterinary pathologist.

### 4.4. Quantitative Analysis of Smooth Muscle Cells Nuclei in the Muscular Layers

For each sample, twelve sections centered in the muscular layers were photographed (objective lens of 10×). For each section, an area of 50 µm × 200 µm (10,000 µm^2^) in the center of the photo was used for nucleus quantification per unit area. Only the nuclei of the SMCs within the test area boundaries and those that touched the lines were counted. SMC nucleus density was expressed as the number of cells per mm^2^ of muscular area.

### 4.5. Quantitative Analysis of Neuronal Nuclei in the Myenteric Plexi

For each sample, three sections stained with H&E were observed, and all myenteric plexi were photographed using 10×, 20×, and 40× objective lenses when needed. The myenteric plexi were then outlined, and their areas were measured. The neurons’ nuclei within all visible sections of the myenteric plexus were counted. Myenteric neuronal density was expressed as the number of cells per mm^2^ of the plexus.

### 4.6. Total GSH and GSSG Quantification

For total GSH (tGSH) and GSSG quantification, 1cm long samples of the proximal duodenum, middle jejunum, distal ileum, cecum, PC, and DC were collected, and 400 μL of perchloric acid 5% (*w*/*v*) was added. The tissues were homogenized and centrifugated at 16,060× *g* for 10 min at 4 °C. The pellets were then saved for protein quantification at −20 °C, and the acidic supernatant was stored at −80 °C until analysis.

The levels of tGSH and GSSG were measured using the DTNB-GSSH reductase recycling assay, following the modified Ellman’s method [78]. Acidic samples were neutralized with 0.76 M potassium bicarbonate and then centrifuged (16,060× *g* for 2 min at 4 °C). The same process was applied to GSH standards ranging from 0 to 15 μM. In 96-well plates, 100 μL of sample was mixed with 65 μL of reagent solution containing NADPH (0.63 mM) and DTNB (3.96 mM) and prepared in phosphate buffer (71.5 mM Na_2_HPO_4_, 71.5 mM NaH_2_PO_4_, 0.63 mM EDTA). The mixture was incubated at 30 °C for 15 min. Subsequently, 40 μL of glutathione reductase (10 U/mL in phosphate buffer) was added, and absorbance readings were taken at 415 nm for 3 min with 10 s intervals, using a Biotek PowerWaxe X spectrophotometer (Charlotte, VT, USA). The tGSH and GSSG levels were normalized to protein levels and expressed as nmol/mg of protein. The GSH/GSSG ratio was calculated using the following formula:

(1) $$\mathrm{GSH}/\mathrm{GSSG}=\frac{tGSH-2\times GSSG}{GSSG}$$

### 4.7. Protein Quantification

The pellets described in the previous section were dissolved in 0.5 M NaOH, and an albumin stock solution was prepared with concentrations ranging from 0.0625 mg/mL to 1 mg/mL. The pellets were homogenized, and protein levels were assessed spectrophotometrically using a microplate reader (Biotek-Powerwave HT^®,^ Charlotte, VT, USA), following the method described by Lowry et al., with measurements taken at a wavelength of 700 nm [79].

### 4.8. Statistical Analysis

The GraphPad Prism 8.1.2 was used for statistical analysis of data. The unpaired Student’s *t* test was used for comparison between 2 experimental groups (CTRL and GK), and data were expressed as mean ± SEM, where *n* refers to the number of experimental animals. The Shapiro–Wilk test was employed to assess the normality of the data. All datasets had *p* > 0.05 and were considered to have passed the normality test. To evaluate histological and oxidative stress data, a two-way ANOVA followed by an unpaired *t* test with Welch’s correction was used to compare the two experimental groups. In all cases, a *p* value of less than 0.05 was used to identify a statistically significant difference.

## Data Availability

Dataset available on request from the authors.

## Supplementary Materials

The following supporting information can be downloaded at: https://www.mdpi.com/article/10.3390/ijms252212115/s1.
