# Supplementary material for: Oxidative Stress and Histomorphometric Remodeling: Two Key Intestinal Features of Type 2 Diabetes in Goto–Kakizaki Rats
Source: Int J Mol Sci. 2024 Nov 12;25(22):12115. doi: 10.3390/ijms252212115 (PMC11594829; doi:10.3390/ijms252212115)
Supplement: Supplementary file 1 [file ijms-25-12115-s001.zip › ijms-3255611-supplementary.pdf]

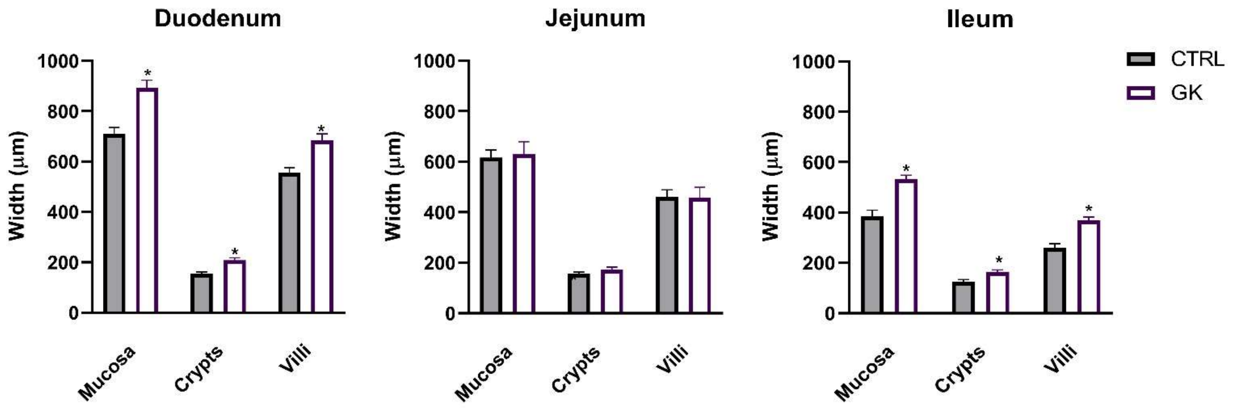

Figure S1. Morphometric analysis of mucosa, differentiated by crypt depth and villus height, of all intestinal segments (duodenum, jejunum, ileum) in control rats (CTRL, n = 5) and GK diabetic rats (GK, n = 6). Results are expressed as mean ± SEM. Comparisons between CTRL and GK groups were made using 2-way ANOVA followed by unpaired t-test with Welch's correction. *$p < 0.05$ indicates significant difference from corresponding control.
